# Supplementary material for: Dynamic PD-L1 Regulation Shapes Tumor Immune Escape and Response to Immunotherapy
Source: Cancers (Basel). 2025 Nov 27;17(23):3803. doi: 10.3390/cancers17233803 (PMC12691448; doi:10.3390/cancers17233803)
Supplement: Supplementary file 1 [file cancers-17-03803-s001.zip › cancers-3936150-supplementary.pdf]

Supplementary Materials: Dynamic PD-L1 Regulation Shapes Tumor Immune Escape and Response to Immunotherapy

Bruce Pell [1,*], Aigerim Kalizhanova [2], Aisha Tursynkozha[3], Denise Dengi[4], Ardak Kashkynbayev[2] and Yang Kuang[4]

1. Supplementary Table S1: Model Comparison for EMT-6 Data

Table S1. Comparative Model Goodness-of-Fit for EMT-6 Data. Residual Sum of Squares (RSS) and Akaike Information Criterion (AIC) for the dynamic ϵ and constant ϵ models. The models were fit to two different datasets: the "7-Mouse Mean" (representing the 87.5% curative phenotype) and the "8-Mouse Mean" (the composite average including the single non-responding mouse found in the high-dose combination therapy).

Model	Dataset	RSS	AIC
Dynamic ϵ	7-Mouse Mean	47,626.20	268.75
Dynamic ϵ	8-Mouse Mean	190,393.58	318.64
Constant ϵ	7-Mouse Mean	142,600.67	261.05
Constant ϵ	8-Mouse Mean	149,931.08	270.90

2. Supplementary Figure S1: Dynamic Model Fit to 8-Mouse Composite Mean

The 2x3 grid plot showing the poor global fit of the 5-parameter dynamic epsilon model and constant epsilon model when they are forced to fit the 8-mouse composite mean.

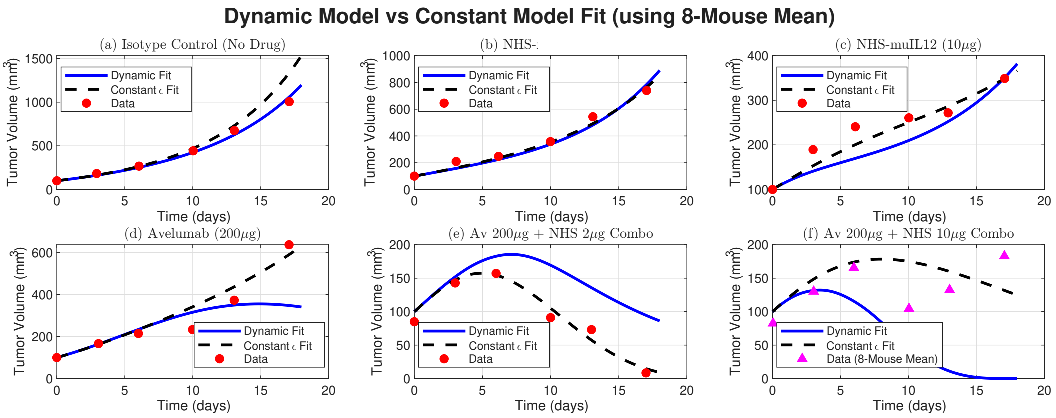

Figure S1. Comparative Model Fits to the 8-Mouse Composite Mean Dataset. Each panel displays the experimental data for one of the six EMT-6 treatment arms. The data for the high-dose combination therapy (f) is the 8-mouse composite mean (magenta triangles), while Modes 1-5 use the 8-mouse mean (red dots) that was used in the manuscript figures. Overlaid are the best-fit simulations from two different models, both of which were fit to this 8-mouse composite dataset: the 5-parameter global dynamic epsilon Model (blue solid line) and the 6-parameter local Constant epsilon model (black dashed line). The resulting poor fits, particularly the global model's (blue line) inability to capture the correct dynamics in the combination arms (d, e, f), demonstrate the difficulty of fitting a single model to a composite average of a bimodal biological response.

3. Supplementary Figure S2: Comprehensive Sensitivity Analysis (Epsilon)

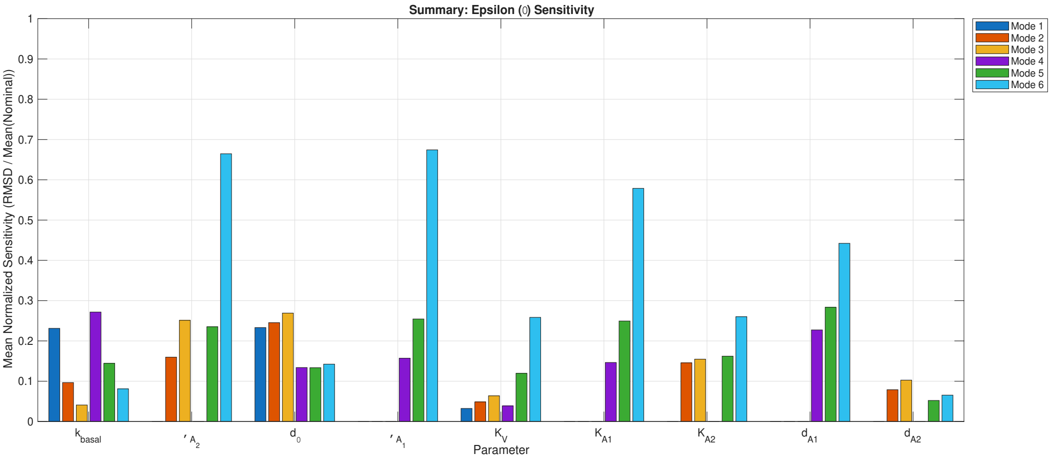

Figure S2. Normalized average sensitivities of the model's ϵ output to a $\pm 25\%$ perturbation in each of the nine key parameters, grouped by treatment mode. The nine parameters tested are the five fitted ϵ-parameters (k_{basal}, α_{NHS}, d_ϵ, α_{Av}, K_V), the two fixed parameters (d_{A_1}, d_{A_2}), and the two key binding constants (K_{A_1}, K_{A_2}).
